# Reduced representation bisulfite sequencing (RRBS) of dairy goat mammary glands reveals DNA methylation profiles of integrated genome-wide and critical milk-related genes

**DOI:** 10.18632/oncotarget.23260

**Published:** 2017-12-15

**Authors:** Xiaoyan Zhang, Sihuan Zhang, Lin Ma, Enhui Jiang, Han Xu, Rui Chen, Qing Yang, Hong Chen, Zhuanjian Li, Xianyong Lan

**Affiliations:** ^1^ College of Animal Science and Technology, Northwest A&F University, Shaanxi Key Laboratory of Molecular Biology for Agriculture, Yangling, Shaanxi 712100, China; ^2^ College of Animal Science and Veterinary Medicine, Henan Agricultural University, Zhengzhou 450002, China

**Keywords:** dairy goat, mammary gland, DNA methylation, reduced representation bisulfite sequencing (RRBS), milk-related genes

## Abstract

DNA methylation (DNAm), a major element of epigenetics, plays critical roles in individual development. Reduced representation bisulfite sequencing (RRBS) is an effective and economical method for analyzing the DNA methylation of a single base. The aims of this study were to determine the DNAm profiles of the methylation contexts (CGs and non-CGs) of lactation and dry periods of goat mammary glands using the RRBS, and to identify potential milk-related genes. The proportion of CG was the highest among all the sequence contexts. The highest CG levels (72.44% to 75.24%) occurred in the 3′ UTR region, followed by the gene body region (61.14% to 65.45%). The non-CG levels were low compared to the CG levels. Bioinformatic analysis demonstrated that the CGs were mainly enriched at high methylation levels (>90%), while non-CGs were enriched at low methylation levels. Methylation levels of 95 and 54 genes in the lactation period were up- or downregulated, respectively, relative to the dry period, such as *PPARα, RXRα* and *NPY* genes. The bisulfite sequencing PCR results showed that the methylation level of goat *PPARα* gene during the lactation period was significant lower than in the dry period, while the methylation level of the *RXRα* gene was lower in the dry period than in the lactation period. Meanwhile, the methylation levels of human *PPARα* and *NPY* genes were significantly higher in MCF-7 than in MCF-10A cells. These findings provide essential information for DNA methylation profiles of goat mammary gland and detect some potential milk-related genes in dairy goats.

## INTRODUCTION

Dairy product is a vital source of nutrition; ruminant milk contains special active proteins, lipids, immunoglobulins, cytokines, antimicrobial peptides, hormones, and growth factors [1, 2]. Compared to bovine milk, goat milk is more digestible, and more easily triggers the body’s innate and adaptive immune systems [3, 4, 5]. However, breeding and milk production of dairy goats are carried out at a lower scale compared to those of the dairy cow. Therefore, dairy goat milk production needs to be more closely monitored in terms of performance and quality.

Several considerable studies have revealed that DNA methylation (DNAm) plays critical roles in mammary gland development and lactation function. Genome-wide methylation patterns in human mammary epithelial cells demonstrate that DNAm participates in the control of human mammary epithelial cell polarity and cellular differentiation [6]. DNAm studies on lactation are gaining increasing attention, suggesting that epigenetics plays an important role in mammary gland development and function [7–9]. However, the specific methylation patterns and genes related to goat mammary gland development and function were rarely reported.

DNAm, the most common epigenetic modification, is involved in diverse processes [10, 11]. CpG islands, which normally remain unmethylated [12], are the most prominent features of DNAm patterns in livestock. The methylation levels CpG islands are generally hypomethylated, and a small number of methylation sites are randomly distributed. The CpG islands of highly expressed genes, such as housekeeping and tissue-specific genes, maintain a low methylation status. DNAm in mammals, mainly targeted in the CG sequence context, mediates gene expression. To date, non-CpG (e.g. CpA, CpT, and CpC) methylations are functional and commonly occur in plant cells; however, there have been few studies in this regard in animal cells. Recently, mammalian cells have been verified to contain non-CpG methylation (CpGm) patterns [13]. A comprehensive analysis of non-CpG methylation across human pluripotent and differentiated cells detected a strong correlation between non-CpG methylation and DNMT3 (DNA (cytosine-5)-methyltransferase 3) expression levels, suggesting that non-CpG methylation may be linked to CpG methylation [14]. Despite the different mechanisms of CG methylation (CGm) and non-CGm, the DNAm of gene promoters is involved in influencing gene expression [15]. There are few studies on DNAm patterns in goats compared to those in humans, cattle, pigs, and sheep [16–20]. Reduced representation bisulfite sequencing (RRBS) is an economical, accurate, and efficient method to obtain DNAm profiles. This method enriches the promoter and CpG island regions by restriction endonuclease digestion [21]. The CpG-rich restriction fragments, after the bisulfite conversion of unmethylated cytosine, are then sequenced. High-resolution detection of the DNAm status and high-efficiency utilization of the sequencing data are achieved [22].

The RRBS method was first proposed in 2005 [22], and was adopted by numerous researchers. Due to the high single-base resolution and acceptable cost, RRBS has become a widely used method for detecting DNAm. In 2008, analyses of genome-scale DNAm profiles in mouse embryonic stem cells, embryonic stem cell-derived neural stem cells, primary neural cells, and eight other primary tissues were generated using RRBS [23], further verifying that RRBS is an effective technology for the analysis of epigenetic profiles.

Herein, we compared the methylation profiles of the lactation and dry periods of goat mammary glands using the RRBS method. We analyzed the differential methylated genes between the two periods to determine some of the important genes associated with lactation or mammary gland development, with the aim of providing essential information regarding the epigenetic regulation of mammary glands in dairy goats.

## RESULTS

### Data filtering and reads alignment

High quality and clean reads were mapped to goat reference genome (NCBI) by BSMAP (version: 2.90) software. Before alignment to the reference genome, the raw reads were filtered, which included trimming the adapter sequences, adapter contamination, and low-quality reads. The average clean reads, which were 23.01 M in the dry period samples and 20.83 M in the lactation period samples, were then used for subsequent analysis (Table 1). The clean reads were mapped to the goat reference genome. Every hit of a single placement with a minimum numbers of mismatches and a clear strand assignment was defined as an unambiguous alignment (uniquely mapped read). Uniquely mapped reads with restriction enzyme cutting sites were used for further analysis. The rate of the clean reads mapped to the reference genome in the four samples was approximately 64%, and the rate of enzyme cutting site uniqueness was greater than 89% (Table 1). We listed the numbers of reads that each cytosine type (CG, CHG and CHH, H represents non-C base) were covered by (Supplementary Table 1). We obtained a large amount of sites for all cytosine types (CG, CHG and CHH) in 3′ UTR, 5′ UTR, CDS, intron, and the 2-kb downstream and 2-kb upstream regions. This result suggests that there was a considerable number of non-CG sequence contexts located in the genomic regions. Furthermore, there were similar CG, CHG, and CHH coverage rates of approximately 47%–60% in each genomic region, suggesting that non-mCG sequence contexts might exist in mammals.

**Table 1:** Data production and reads alignment

Sample	Insert size	Read length	Clean reads	Clean base	Mapped reads	Map rate	Enzyme cutting reads	Enzyme cutting rate
D1	0-250	100 bp	21.52 M	3.67 Gb	16.00 M	74.35 %	14.33 M	89.56 %
D2	0-250	100 bp	24.50 M	3.62 Gb	15.27 M	62.33 %	13.73 M	89.91 %
M1	0-250	100 bp	20.14 M	2.68 Gb	12.02 M	59.68 %	10.77 M	89.60 %
M2	0-250	100 bp	21.52 M	2.84 Gb	13.16 M	61.15 %	11.81 M	89.74 %

**D1** and **D2** are the samples of goat dry period mammary glands; M1 and M2 are the samples of goat lactation period mammary glands; Enzyme cutting rate: the percentage of unique reads which have enzyme cutting site.

### Cumulative distribution and proportion of CG, CHG, and CHH sequence contexts

**Figure 1:**
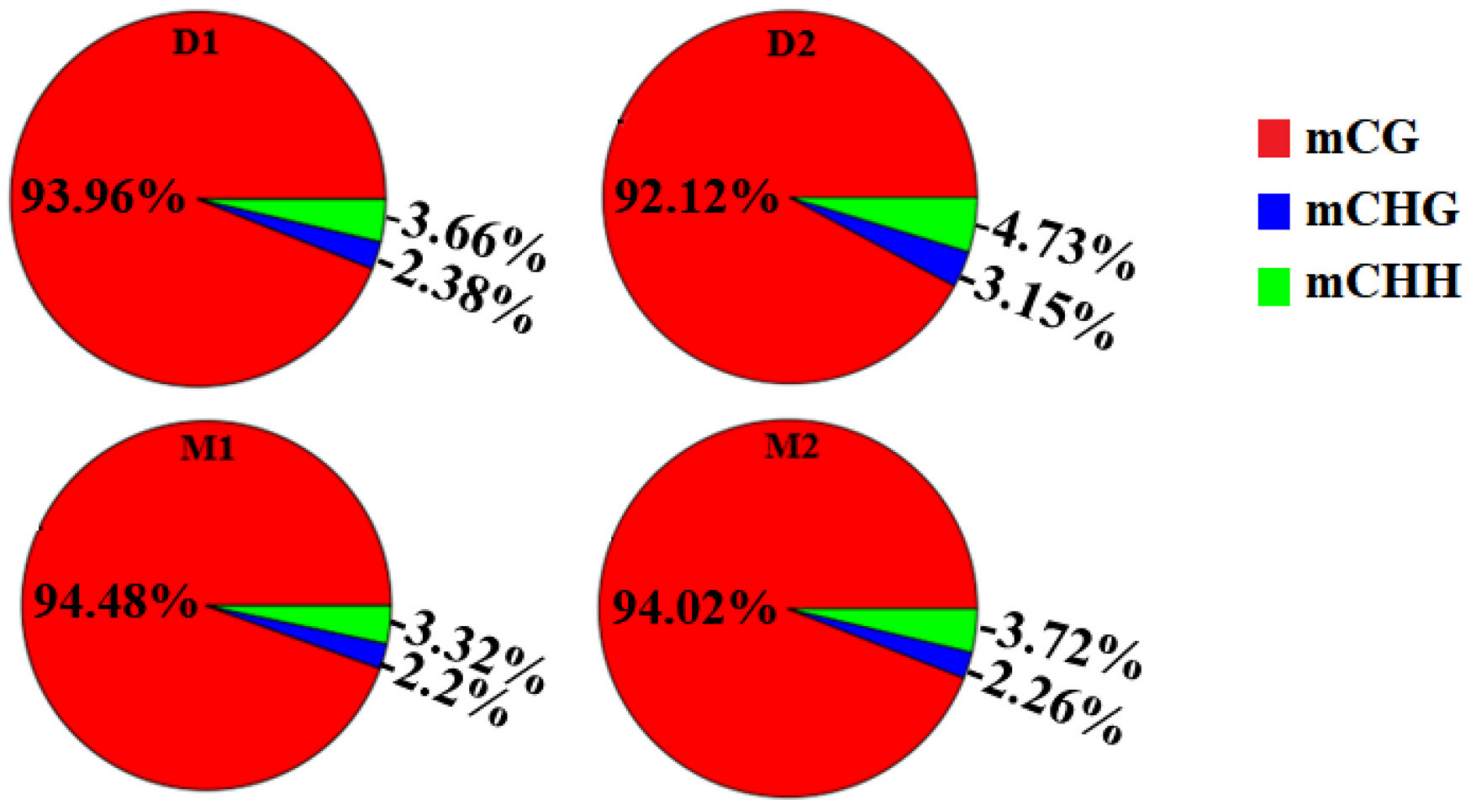
Proportion of different methyl-cytosine patterns in analyzed goat genome of lactation period and dry period mammary glands Note: **D1** and **D2** are the samples of goat dry period mammary glands; **M1** and **M2** are the samples of goat lactation period mammary glands.

**Figure 2:**
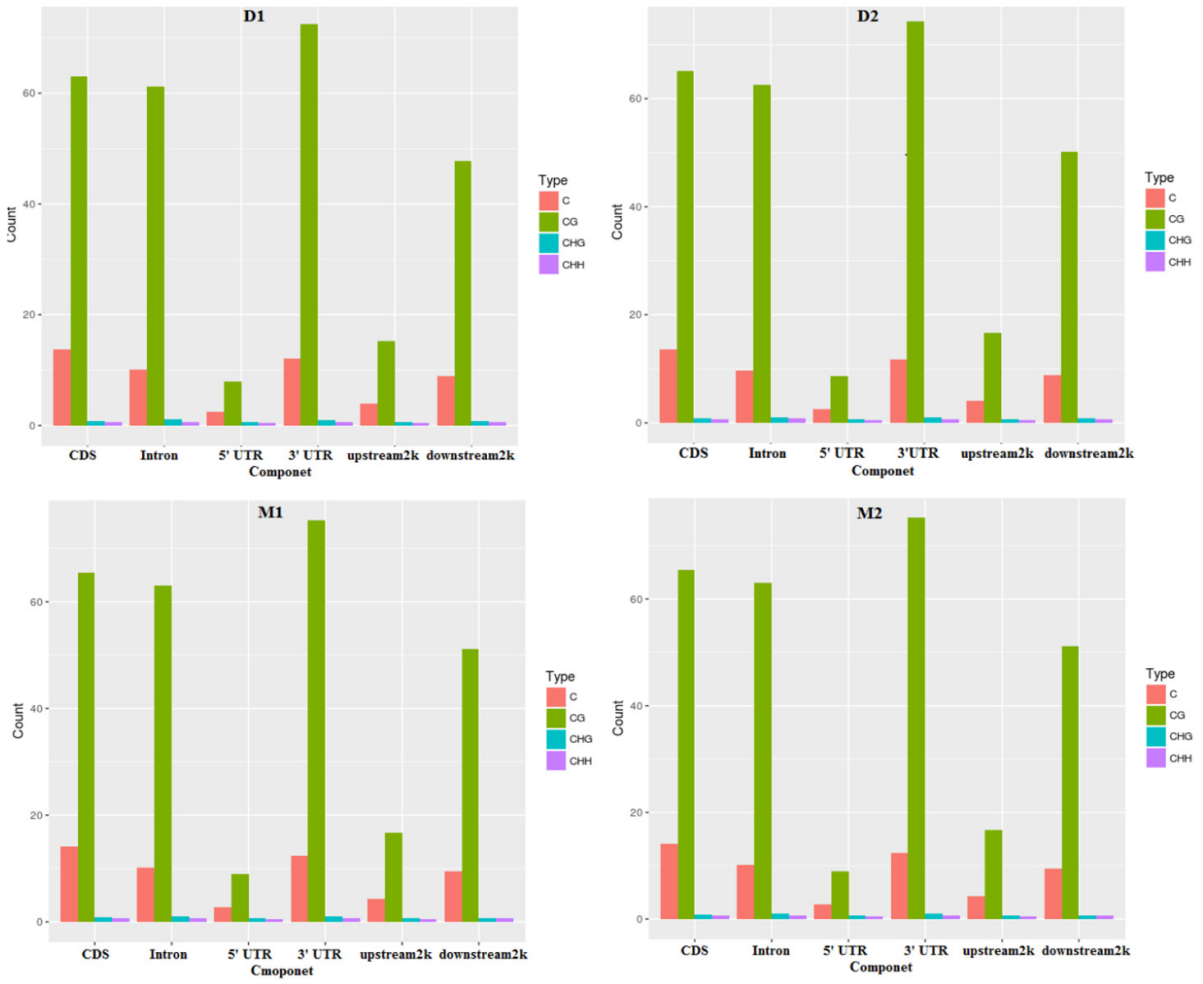
The different kinds of methyl-cytosines distribution in different components of the genome in two periods Note: **D1** and **D2** are the samples of goat dry period mammary gland; **M1** and **M2** are the samples of goat lactation period mammary gland.

In this study, methylated cytosine bases distributed across the genomes contained three forms: mCG, mCHG, and mCHH. The amount and proportion of methylcytosines in different components of the genome of two periods were similar (Table 2 and Figures 1-2). Methylcytosine identification was according to the method and correction algorithm of Lister [24]. The proportion of mCG was higher than the other two sequence contexts, in both the lactation and dry periods (Figure 1). For the different regions, mCG in the 3′ UTR was the highest, followed by in the gene body (CDS and intron) region, while the 5′ UTR, 2-kb upstream region and 2-kb downstream region were hypomethylated. Moreover, the methylation levels of CHG and CHH in all regions were low (Supplementary Table 2).

**Table 2:** The amount and proportion of different types of methylcytosines in analyzed goat genome of lactation and dry periods mammary glands

Sample	Pattern	mCG	mCHG	mCHH
D1	Number	121365	3071	4722
	Proportion (%)	93.96	2.38	3.66
D2	Number	153746	5249	7892
	Proportion (%)	92.12	3.15	4.73
M1	Number	107593	2501	3782
	Proportion (%)	94.48	2.20	3.32
M2	Number	93185	2244	3692
	Proportion (%)	94.02	2.26	3.72

**D1** and **D2** are the samples of goat dry period mammary glands; **M1** and **M2** are the samples of goat lactation period mammary glands.

**Figure 3:**
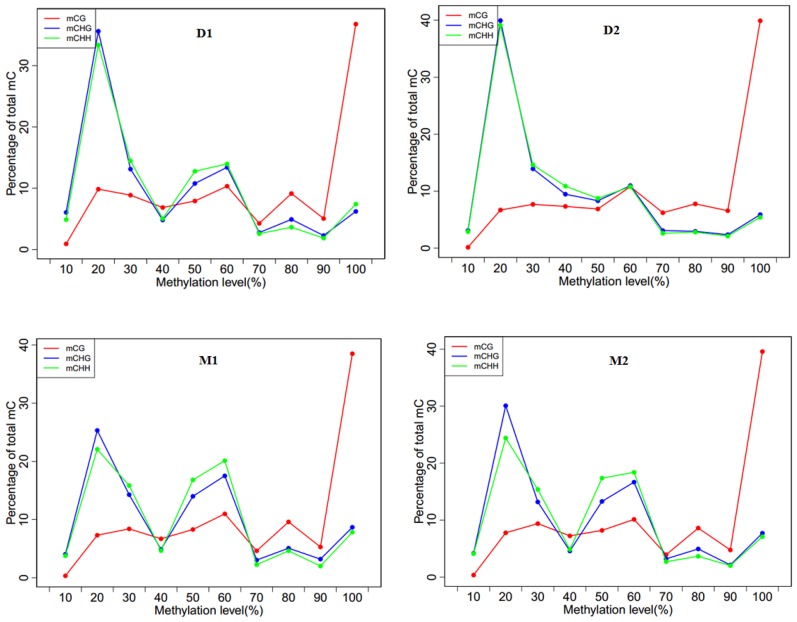
Methylation level distributions for different methyl-cytosine patterns in analyzed goat genome of lactation period and dry period mammary glands Note: **D1** and **D2** are the samples of goat dry period mammary glands; **M1** and **M2** are the samples of goat lactation period mammary glands; The horizontal axis represented methylation level of methyl-cytosines with 10 percentage interval while the vertical axis indicated the percentile distribution; The “mCG” means methylated CG, “mCHG” and “mCHH” as the same.

The mCGs were enriched at a high methylation level (>90%), and the percentage of methylated CG was rapidly increased to above 30%. Both non-CG sequence contexts were enriched only at a low methylation level (Figure 3). In the dry period, the percentages of mCHG and mCHH were increased and then decreased below 40% and retained a low methylation percentage. However, in the lactation period, the percentages of mCHG and mCHH were represented as a wavy line. The heat map indicated that the average methylation level of cytosine in CpG was inversely proportional to the CpG density patterns (Supplementary Figure 1).

### GO enrichment analysis based on differentially methylated region (DMR) related genes

**Figure 4:**
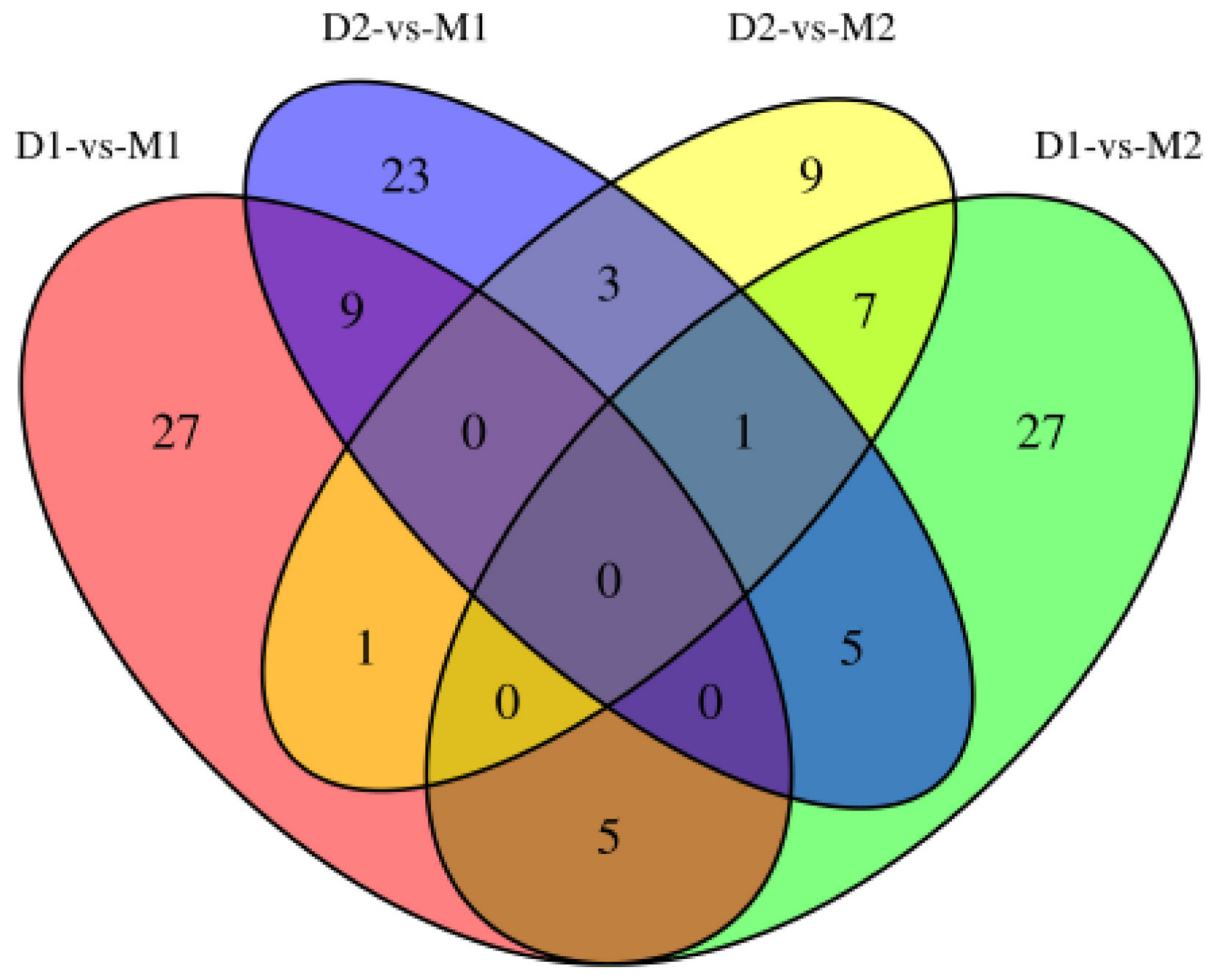
Venn diagram for DMR-related genes of two periods Note: **D1** and **D2** are the samples of goat dry period mammary glands; **M1** and **M2** are the samples of goat lactation period mammary glands; DMR: differentially methylated region.

**Figure 5:**
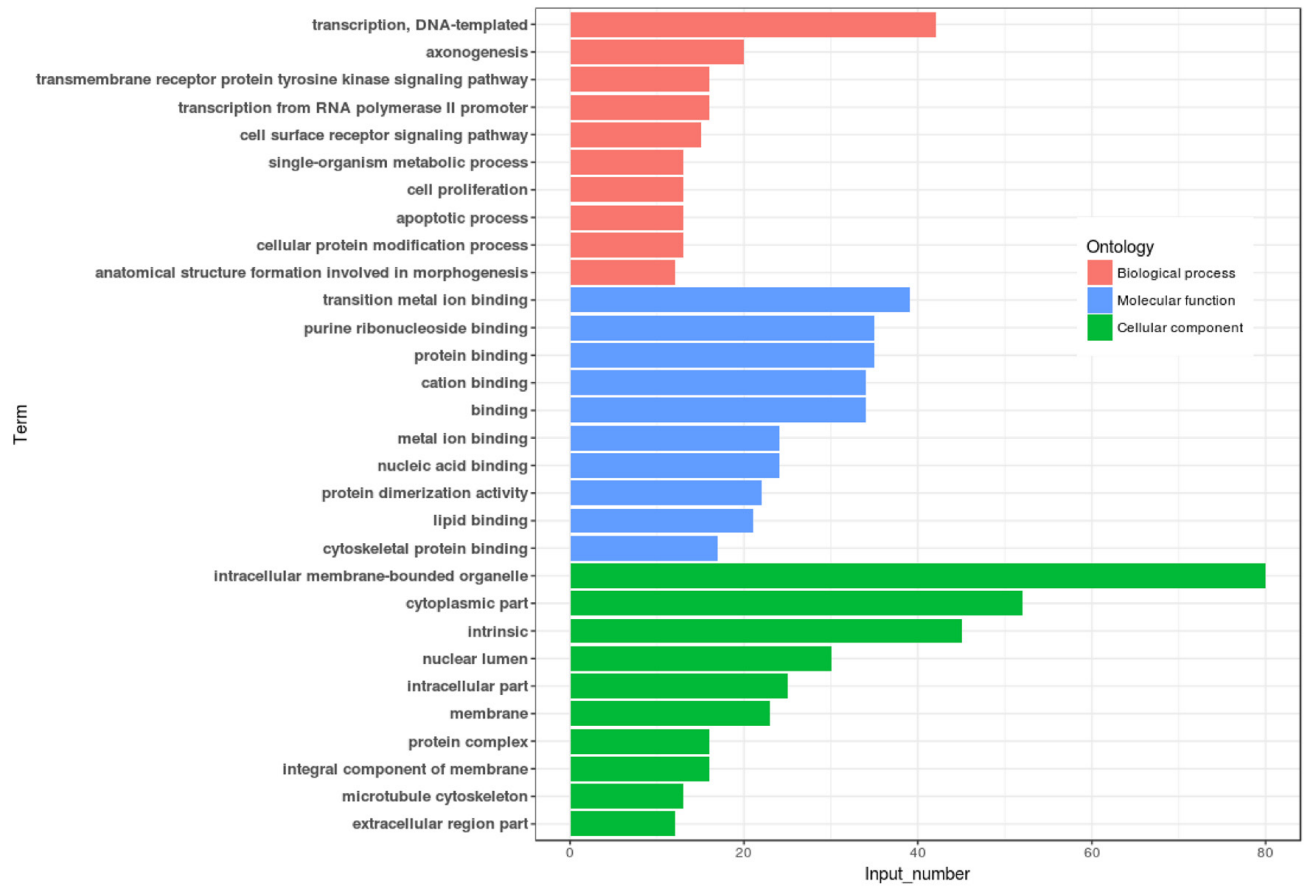
Go annotation of DMR related genes with top ten enrichment numbers covering domains Note: DMR: differentially methylated region.

Differential methylation analysis revealed that the methylation levels of 95 genes were upregulated, while 54 genes were downregulated in the lactation period, relative to the dry period (Table 3 and Figure 4). GO functional analysis, based on DMR-related genes, provided a GO functional classification annotation for DMR-related genes. Detection of DMR-related genes was contributed by identifying potential genes that were associated with mammary gland development and lactation process. All differently expressed genes were mapped to each term of the Gene Ontology database (http://www.geneontology.org/), and the gene numbers of each GO term were calculated. A hypergeometric test was used to determine significantly enriched GO terms of the DMR-related genes compared to the genome background, and the corrected-*P* value ≤ 0.05 was used as a threshold. GO terms fulfilling this condition were defined as significantly enriched GO terms of DMR-related genes. Through this analysis, we were able to recognize the main biological functions that DMR-related genes participated in (Figure 5). Further, we detected a few genes associated with mammary gland development and lactation, such as *peroxisome proliferator-activated receptor α* (*PPARα*), *neuropeptide Y* (*NPY*) and *retinoid X receptor α* (*RXRα*). The methylation levels of *PPARα* and *NPY* genes were downregulated in the lactation period, while those of *RXRα* gene was upregulated in the lactation period, compared to the dry period.

**Table 3:** DMR related genes between lactation period and dry period mammary glands

	D1-vs- M1	D2-vs- M1	D1-vs- M2	D2-vs- M2
**dowmstream2k.down**	PXDN	EFNA5	CDK14	SCN1B
	GSC	PXDN	APC2	MRPL18
	PSD3	LTK	SCN1B	
	ANAPC2	TK1	PSD3	
		LOC102182829	SMG5	
		TCF7L2	PPARA	
**dowmstream2k.up**	F7	CTBP1	EFNA5	MTIF3
	KIAA1257	ATPAF2	TSSC1	
	C21H14orf79	FIS1	MRPL18	
**genebody.down**	KIAA1522	B3GNT5	MTIF3	EFNA4
	HOXC4	KIAA1522	LOC102182829	GBX1
	ICAM4	EFNA4	RNF223	RGS3
	LOC102187872	GRM1	EFNA4	PLAGL1
	RXRA	NRDE2	NPY	RBM38
	LOC102186703	RXRA	MSX1	TEAD2
	GRIK4	RPH3A	CPZ	FMNL1
	CASZ1	TEAD2	GPN1	C102179837
	SLC17A7	TOM1L2	LOC102177267	
	TOM1L2	LHFPL4	MMD	
	LOC102188264	LOC102188264	POLDIP2	
	LOC102172305	TRPM5	ACAP1	
	CHCHD6		FMNL1	
**genebody.up**	C7H19orf60	SYT11	LOC102172305	SYT11
	LOC102188874	NPY	EHBP1L1	ARHGAP27
	LOC102189337	MSX1	DLX6	
	NYAP1	KHSRP	C7H19orf60	
	MTG1	POLDIP2	MISP	
		NYAP1	CRYAB	
		MTG1	SAMD14	
**upstream2k.down**	GPBAR1	LOC102177294	MYO15B	KRT80
	ARHGAP30	INSL3	NUDT8	MAB21L1
	INSL3	ENTPD5	SDHB	SLC39A13
	ENTPD5	PRRX1	TNIP2	ZNF750
	BCAS4	ZNF19	NBEA	ALDH1L1
	KCTD10	UROC1	SLC39A13	SERPINB1
	LOC102173495	ALDH1L1	LOC102173495	
	CDC42EP4		ZNF750	
	IGF2		TOPAZ1	
**upstream2k.up**	POLR3GL	PKN3	BMP6	TNS1
	KRT80	RBBP8NL	C7H19orf67	PNLDC1
	KCNT1	FCGBP	GSN	
	LZTS3	EVPL	PNLDC1	
	SLIT3	PRSS45	CCDC187	
	ZNF394	DTX1	LAMB3	
	LOC102191489		LMNA	
	LMNA			

**D1** and **D2** are the samples of goat dry period mammary glands; **M1** and **M2** are the samples of goat lactation period mammary glands; DMR:differentially methylated region.

### Bisulfite sequencing PCR (BSP) of goat *PPARα* and *RXRα* genes and human *PPARα* and *NPY* genes

In order to verify the accuracy of RRBS and to determine the key genes that function through methylation modification, *PPARα*, *RXRα* and *NPY* genes were chosen to perform the experimental study involvinig in dairy goat mammary glands and cell lines. We intended to detect the methylation levels of the above genes from both organizational and cellular consideration, but there was no mature and commercial goat mammary epithelial cell line; therefore, we used the human mammary epithelial cell line. The homology of *PPARα*, *RXRα* and *NPY* genes between human and goat was relatively high (Supplementary Figure 2 and Table 4); therefore, we were able to detect some of these related genes in human cell lines.

**Table 4:** Pairwise sequence alignment of six species *PPARα*, *RXRα* and *NPY* genes

Amino acid sequences^1^	Identity of two aligned sequences (%)^2^				
***PPARα***	**1**	**2**	**3**	**4**	**5**
1. ALZ41704.1 Capra hircus (goat)					
2. ADW20209.1 Ovis aries (sheep)	78.0%				
3. AAI16040.1 Bos taurus (cattle)	77.0%	98.9%			
4. NP_001007140.2 Homo sapiens (human)	70.4%	94.0%	94.5%		
5. CAA39574.1 Sus scrofa (pig)	71.2%	94.3%	94.5%	94.4%	
6. AAH53489.1 Mus musculus (mouse)	67.1%	90.4%	90.9%	92.3%	91.0%
***RXRα***	**1**	**2**	**3**	**4**	**5**
1. ALZ41704.1 Capra hircus (goat)					
2. ADW20209.1 Ovis aries (sheep)	99.4%				
3. AAI16040.1 Bos taurus (cattle)	99.4%	100.0%			
4. NP_001007140.2 Homo sapiens (human)	99.4%	100.0%	100.0%		
5. CAA39574.1 Sus scrofa (pig)	99.4%	100.0%	100.0%	100.0%	
6. AAH53489.1 Mus musculus (mouse)	98.8%	99.4%	99.4%	99.4%	99.4%
***NPY***	**1**	**2**	**3**	**4**	**5**
1. ALZ41704.1 Capra hircus (goat)					
2. ADW20209.1 Ovis aries (sheep)	100.0%				
3. AAI16040.1 Bos taurus (cattle)	99.0%	99.0%			
4. NP_001007140.2 Homo sapiens (human)	93.8%	93.8%	94.8%		
5. CAA39574.1 Sus scrofa (pig)	92.8%	92.8%	93.8%	92.8%	
6. AAH53489.1 Mus musculus (mouse)	89.7%	89.7%	90.7%	92.8%	87.6%

The pairwise sequence alignment (PROTEIN) was conducted using EMBOSS Needle method (using Needleman-Wunsch algorithm): http://www.ebi.ac.uk/Tools/psa/emboss_needle/.

**Amino acid sequences**^1^: The gene amino acid sequence name of each species was shown as: Number + Protein_id + The Latin name of the species + (Species);

**Identity of two aligned sequences (%)**^2^: The identity of the two aligned sequences was shown as percentage.

**Figure 6:**
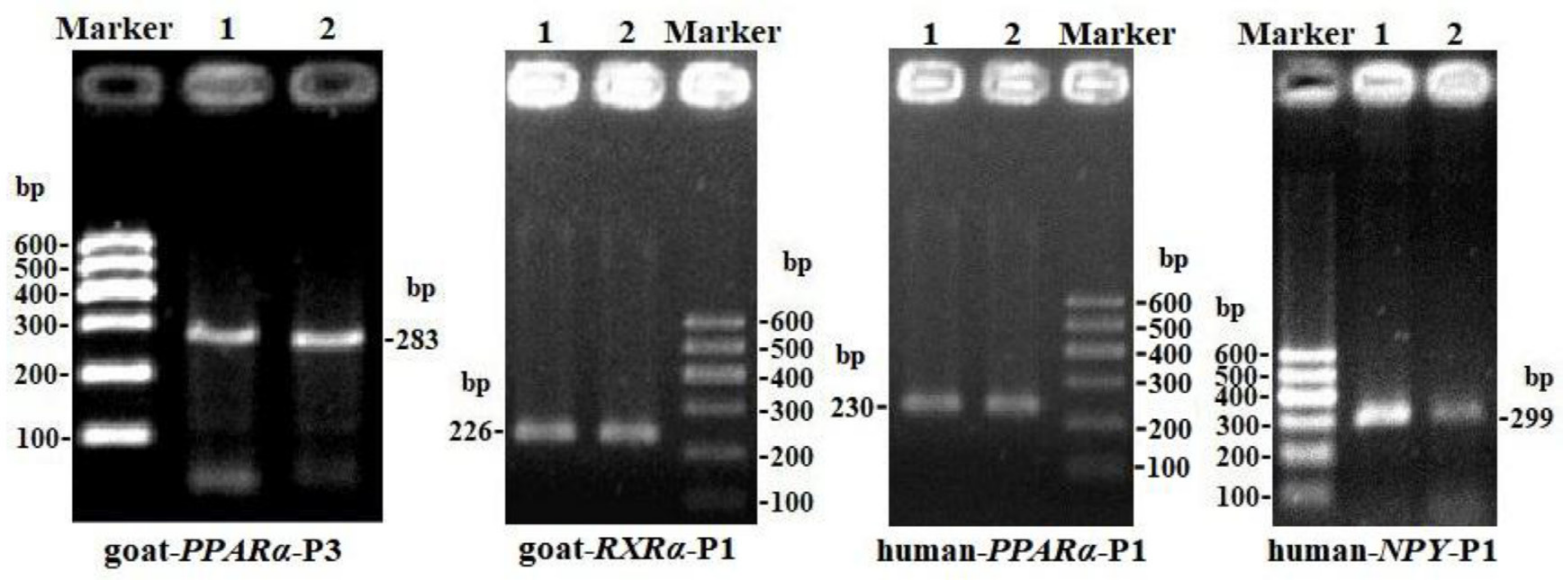
PCR electrophoresis diagrams of CpG islands of goat *PPARα*-P3 and *RXRα*-P1 and human *PPARα*-P1 and *NPY*-P1 genes

**Figure 7:**
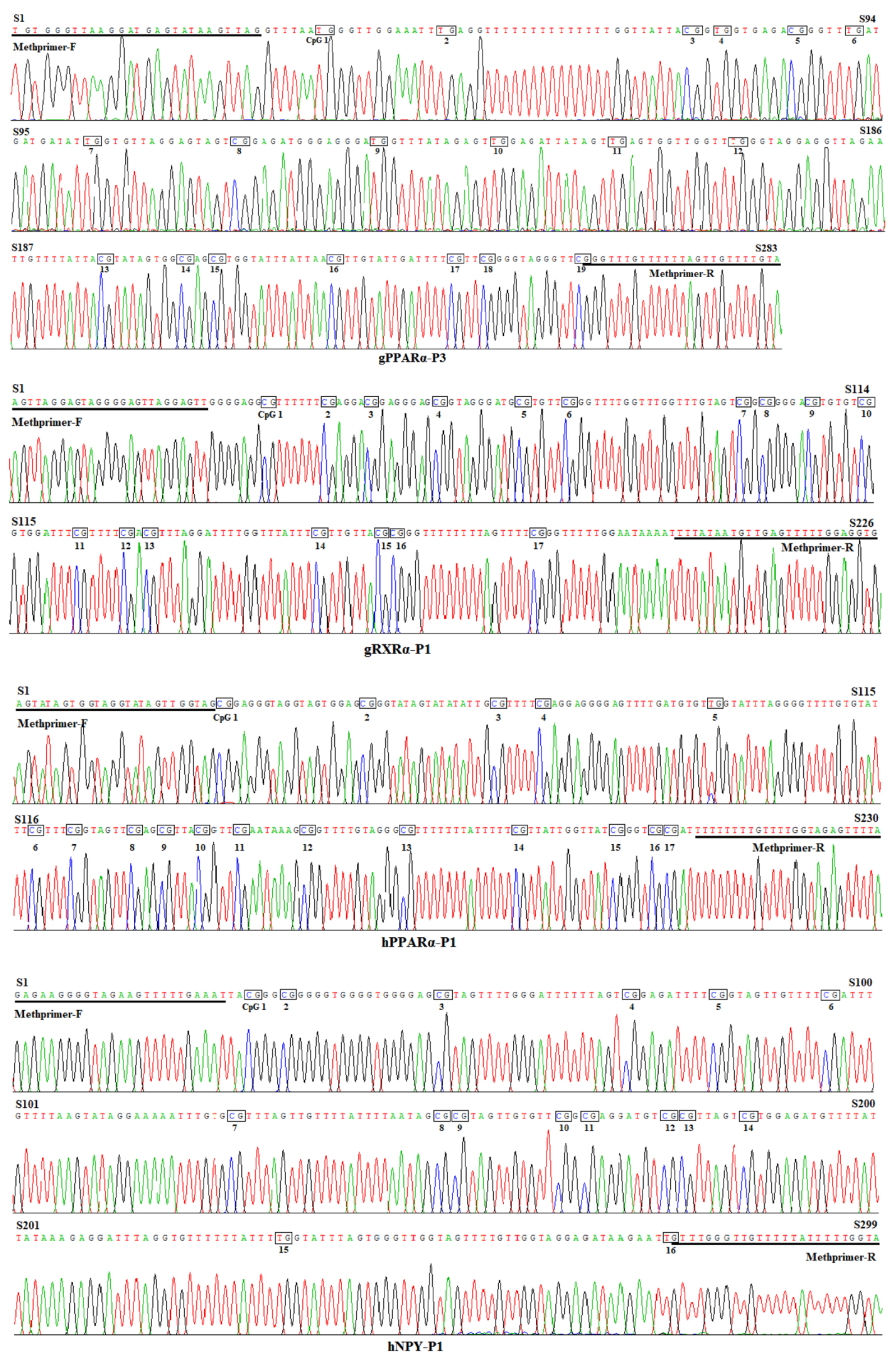
Bisulfite sequencing maps of CpG islands of goat *PPARα*-P3 and *RXRα*-P1 and human *PPARα*-P1 and *NPY*-P1 genes

**Figure 8:**
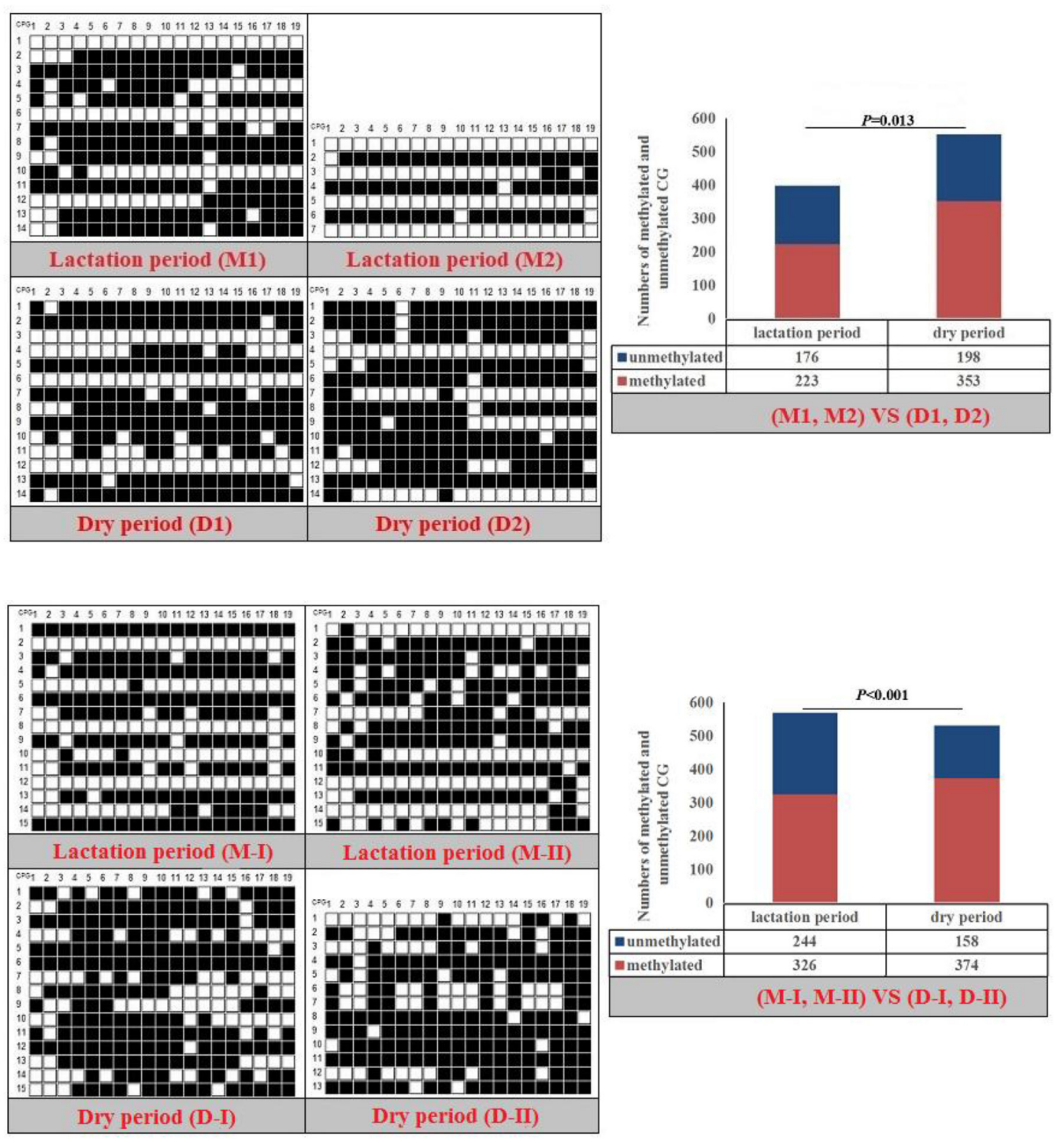
Methylation patterns and significant difference of goat *PPARα*-P3 CpG island of between lactation and dry periods (**Note:** Black and white rectangles represented methylated CG and non-methylated CG loci, respectively).

**Figure 9:**
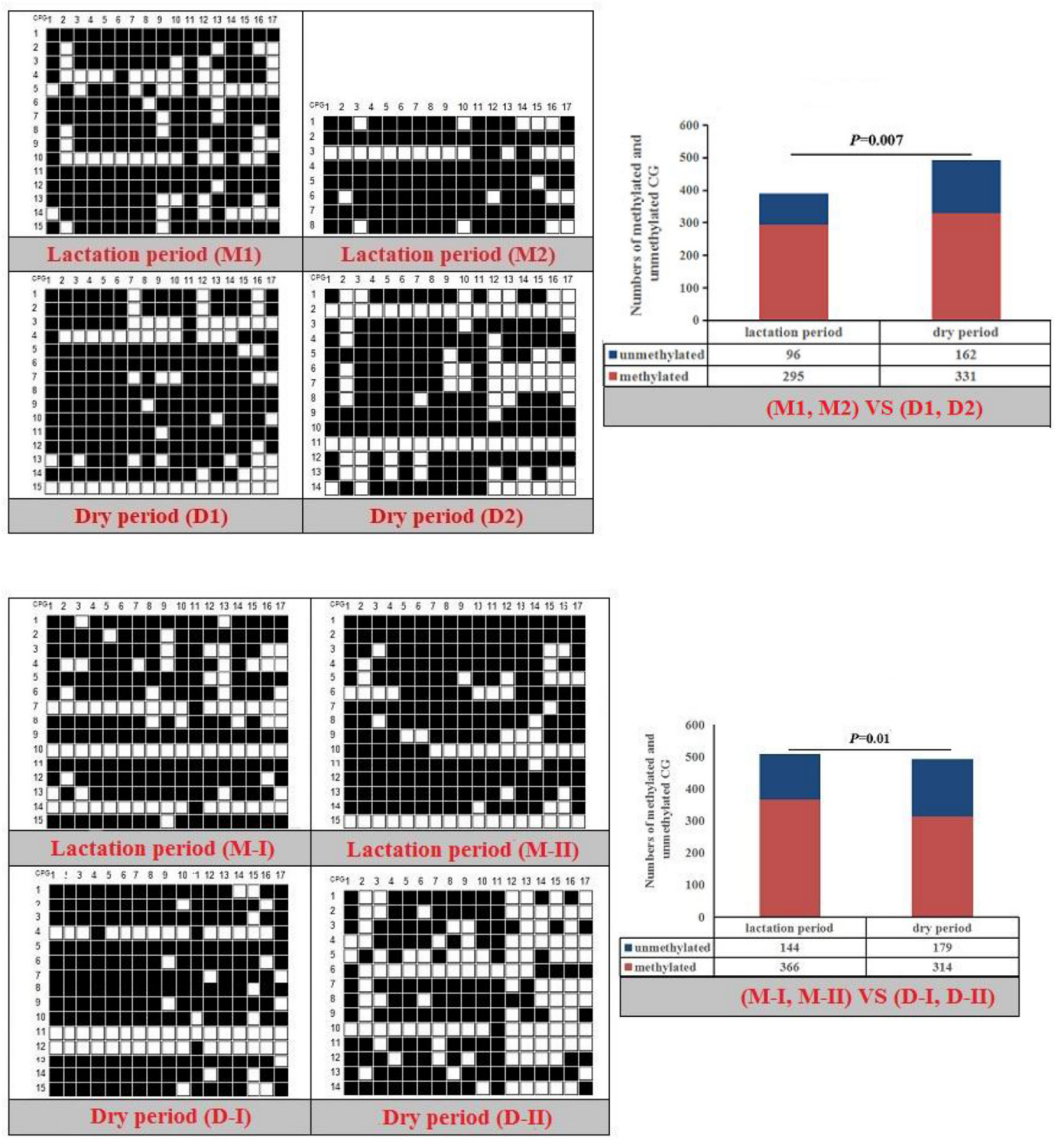
Methylation patterns and significant difference of goat *RXRα*-P1 CpG island of between lactation and dry periods

**Figure 10:**
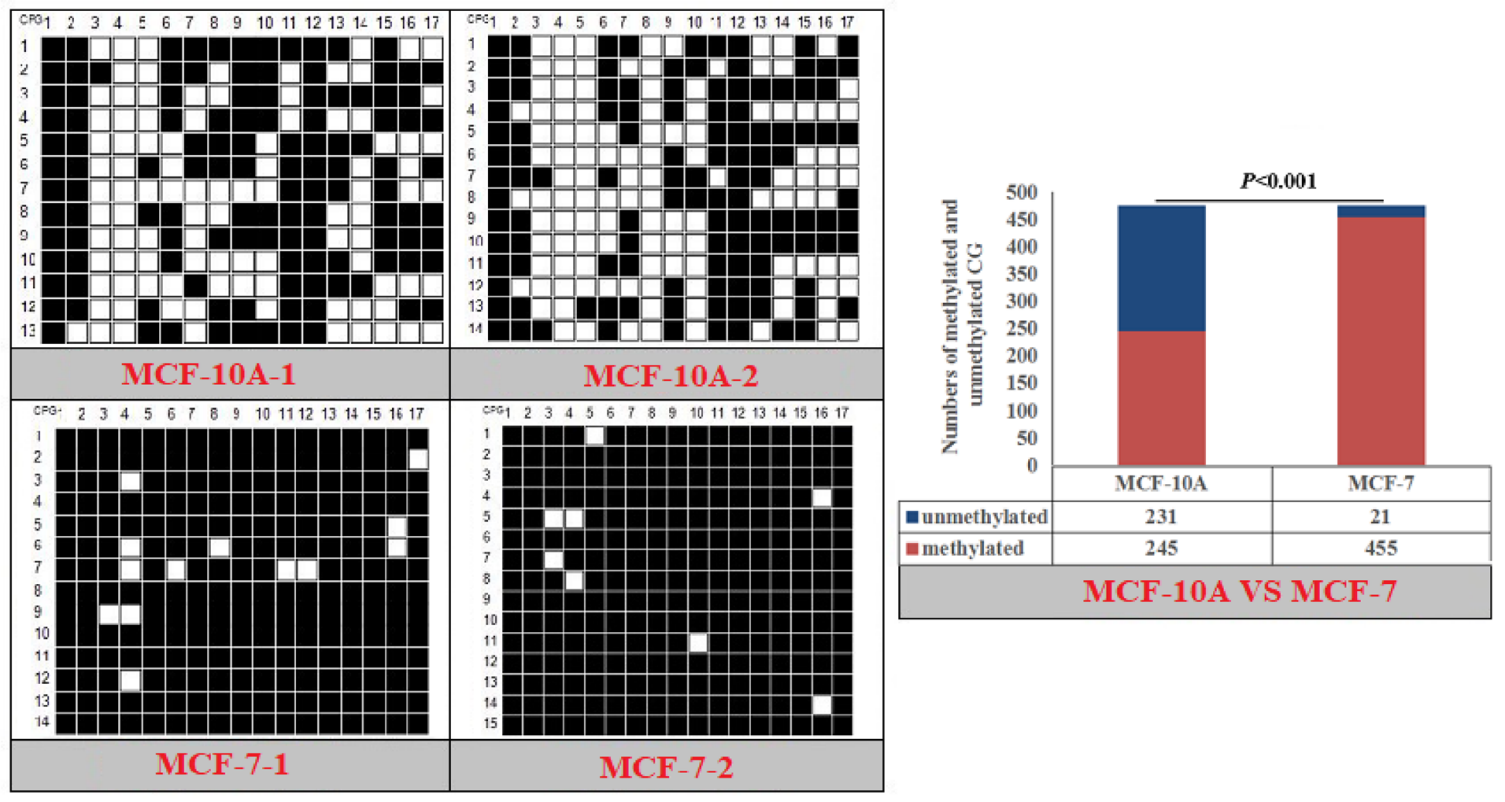
Methylation patterns and significant difference of human *PPARα*-P1 CpG island of between MXF-10A and MCF7

**Figure 11:**
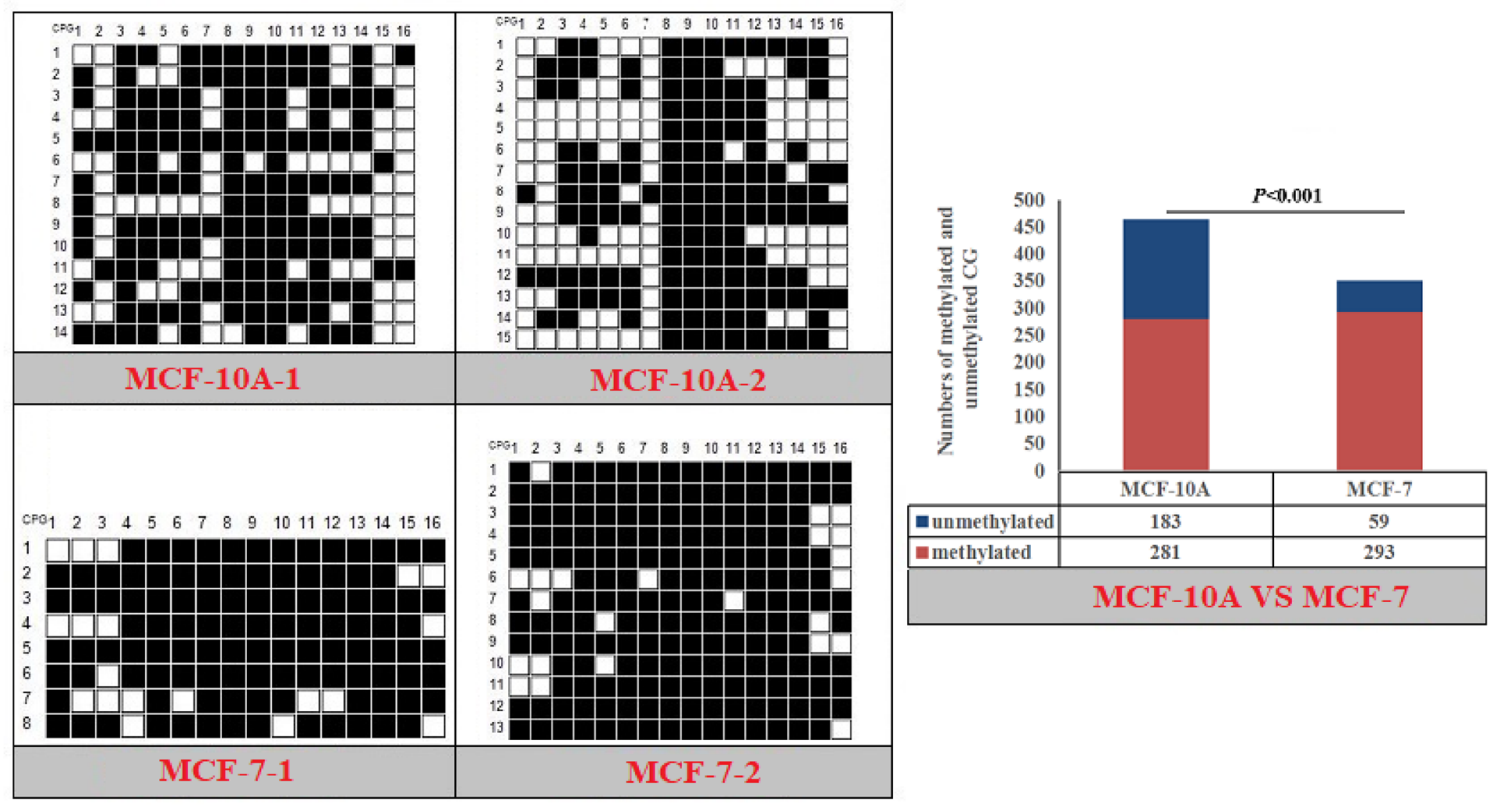
Methylation patterns and significant difference of human *NPY*-P1 CpG island of between MXF-10A and MCF7

The CpG islands and methylation primers of the 5′ flanking, gene body, and 3′ flanking regions of goat *PPARα* and *RX*Rα genes and human *PPARα* and *NPY* genes were predicted and designed for BSP (Supplementary Figure 3-6 and Table 5). DNA treated with bisulfite was used as a template to amplify the CpG islands of goat *PPARα*-P3 and *RXRα*-P1 genes and human *PPARα*-P1 and *NPY*-P1 genes, of which the products were 283, 226, 230 and 299 bp, respectively (Figure 6 and Supplementary Figure 7). After TA cloning, 15-20 positive clones were selected and sequenced for each sample to identify the methylation status (Figure 7). The methylation patterns of all genes were displayed in White-Black Rectangle, wherein black and white rectangles represented methylated CG and non-methylated CG loci, respectively (Figure 8-11).

**Table 5:** BSP primers design for CpG islands of goat and human genes

Primer names	Primer sequences(5′→3′)	Product sizes	CpGs numbers	Posotion
gPPARα-P1	F: GTTGGTATTTGGGGTTTTGTGTR: ACCTACCCTCCCCCAATAAATA	195 bp	13	5′- flanking region
gPPARα-P2	F: TTTGTGGTGTGTTAGGTGATATTTTR: CACTAAACAAACCCAACTTTTATTAC	223 bp	6	5′- flanking region
gPPARα-P3	F: TGTGGGTTAAGGATGAGTATAAGTTAGR: TACAAAACAACTAAAAAACAAACCC	283 bp	19	3′- flanking region
gRXRα-P1	F: AGTTAGGAGTAGGGGAGTTAGGAGTTR: CACCTCCAAAAACTCAACATTATAAA	226 bp	17	intron 1
gRXRα-P2	F: GTGTGGGAAGGAAAGTAGTTTTATTTR: CCTAACCATTACCCAACACTAACC	228 bp	17	intron 1
gRXRα-P3	F: TTATTTGGTTTAAAGTGAAATTTTTR: AACTTAACTACAACACAACCCAC	177 bp	11	intron 3
hNPY-P1	F: GAGAAGGGGTAGAAGTTTTTGAAATR: TACCAAAAATAAAAACAACCCAAAC	299 bp	16	5′- flanking region
hNPY-P2	F: AGGGAGAAAAGTGATTTAGTAGGAAGR: CCAAAAATAACTAACACCACCTTAC	204 bp	16	intron 3
hPPARα-P1	F: AGTATAGTGGTAGGTATAGTTGGTAGR: TAAAACTCTACCAAAACAAAAAAAA	230 bp	17	5′- flanking region
hPPARα-P2	F: AGGTTGGTTTAGGAGTTTTGTGTAGR: TCCAAAAAATTCTACCTCCCTTATAT	186 bp	7	3′- flanking region

### DNAm comparison of entire CpG islands and each CpG dinucleotide locus

Fisher’s exact test showed that methylation differences existed in the goat *PPARα*-P3 and *RXRα*-P1 genes from mammary glands between the lactation and dry periods, and also in human *PPARα*-P1 and *NPY*-P1 genes between the MCF-10A and MCF-7 cells (Figure 8-11 and Table 6). The results showed that the methylation level of the entire CpG islands of the goat *PPARα* gene in the lactation period was significant lower than that in the dry period (*P* < 0.05 or *P* < 0.01), which was in accordance with the RRBS results. There was a significant higher methylation level in the CpG islands of the goat *RXRα* gene in the lactation period than that in the dry period, which was also in accordance with the RRBS results (*P* < 0.05 or *P* < 0.01). On the other hand, the methylation percentages of the human *PPARα* and *NPY* genes had a significantly high level in the MCF-7 cells compared to the MCF-10A cells (*P* < 0.05 or *P* < 0.01) (Figure 8-11 and Table 6).

**Table 6:** Methylation percentages of entire CpG islands of goat mammary gland tissues and human mammary gland cells

Goat	M1 (%)	M2 (%)	D1 (%)	D2 (%)	*P* value	M-I (%)	M-II (%)	D-I (%)	D-II (%)	*P* value
gPPARα-P3	62.78	42.11	60	63.86	*P*=0.013	56.14	58.25	69.82	70.85	*P*<0.01
gRXRα-P1	73.33	79.41	73.33	60.50	*P*=0.007	67.06	76.47	73.73	52.94	*P*=0.01
**Human**	**MCF-10A-1 (%)**		**MCF-10A-2 (%)**			**MCF-7-1 (%)**	**MCF-7-2 (%)**			***P* value**
hPPARα-P1	52.94		50			94.54	96.64			*P*<0.001
hNPY-P1	64.29		57.08			84.38	82.59			*P*<0.001

**D1** and **D2** are the samples of goat dry period mammary gland; M1 and M2 are the samples of goat lactation period mammary gland.

**Note:** The mammary gland tissues of dry period (D1 and D2) and lactation period (M1 and M2) were the samples used for RRBS sequencing. The mammary gland samples of dry period (D-I and D-II) and lactation period (M-I and M-II) were different from the sequence samples.

The methylation patterns and percentages of the each CpG dinucleotide locus of P*PARα*-P3 and *RXRα*-P1 genes in goat mammary gland tissues, and *PPARα*-P1 and *NPY*-P1 genes in human MCF-10A and MCF-7 cells, were calculated (Supplementary Figure 8-9 and Supplementary Table 3-4). The results showed that some of the CpG-dinucleotide loci had significant differences between the two periods or the two types of cells.

## DISCUSSION

Bisulfite sequencing can detect the whole genome DNAm level of each base position, and is an ideal technology to study DNAm of different species and tissues [25–27]. However, accurate assessment of the methylation level of each base position requires high coverage genome-wide DNAm sequencing of whole genomes. This study used RRBS sequencing because of its simple operation, small data requirement, and low cost. RRBS can achieve single-base analysis of genomic DNAm, and is suitable for detecting DNAm differences among tissues or environments. RRBS has been used to study the methylation profiles of bovine [28], pig [29, 30] and human breeds [31], especially in cancer [32].

Mammalian methylation patterns are conservative, and most of them occur in the CG motif [33–35]. In the present study, the proportions of mCG were the highest in both lactation and dry periods, compared to non-CGm. The methylation status of CG, CHG, and CHH differed between species, and there were differences in the spatial, temporal, and physiological dimensions of methylated cytosines within a single organism. The methylation levels of CG were highest in the 3′ UTR region, followed by the gene body region (CDS and intron), while the 5′ UTR, 2-kb upstream, and 2-kb downstream regions were hypomethylated. These results were also observed in rats and humans [36, 37]. The methylation pattern in the transcription area was lower than that in the upstream and downstream region in plants [38, 39]. Additionally, hypermethylation was found in the 3′ UTR region in the present study. Post-transcriptional regulation is the important regulatory mechanism for gene expression, in addition to transcriptional regulation. RNA-binding proteins combine with specific mRNA 3′ UTRs to determine mRNA stability, gene expression localization, and translation [40, 41]. The hypermethylation of 3′ UTR may therefore participate in mRNA stability.

The results showed that the numbers of mCG, mCHG, and mCHH genes in the dry period were higher than that in the lactation period. A considerable part of current research shows that mammary gland development and lactation processes are regulated by methylation. The DNAm level in the lactating mammary gland is lower than that in the liver [42]. The milk protein related genes are detected by hypomethylation only in the lactation period, and by hypermethylation in other periods [7]. The decline of milk protein gene expressions may be regulated by DNAm during mammary involution in dairy cows [9]. Ziller et al (2011) analyzed the methylation characteristics of human pluripotent stem cells and differentiated stem cells, and confirmed that mature cells displayed non-CpG methylation patterns. Compared with embryonic stem cells, the percentage of methylation in non-mature cells was lower, which indicated that the non-CpGm might also have tissue specificity. In addition, the paper also argued that there was a close link between CpGm and non-CpGm [14]. The results of the present study showed that the methylation distributions of CpG were the opposite of the non-CpG distributions. We found that non-CpGs were enriched at the hypomethylation regions, which was consistent with the study of cattle somatic tissues [29]. However, the functions of non-CpG genes have not received much attention, and remain unclear in animals until this study. Our preliminary results implied that the non-CpG genes may function in gene expression and tissue development.

During the parturition process, the pituitary gland secretes a large amount of prolactin to stimulate the mammary gland, initiating normal secretion activity. The prolactin expression then declines, stopping lactation and initiating the dry period. This phenomenon occurs because it is more of a physiological change, in which methylation is essential. Significantly different methylation profiles between lactation and dry periods were observed. Studies showed that rat lactating mammary glands can synthesize polyunsaturated fatty acids with the transcription factor *PPARα* [43, 44]. In the present study, the methylation degree of the *PPARα* gene was downregulated in lactation, while the degree of methylation of the *RXRα* gene was upregulated in the lactation period. *PPARα* plays vital roles in the anabolism and catabolism of fatty acids [45]. It is a member of the steroid hormone receptor family, and its binding with ligands causes a conformational change, forming the *PPAR-α-RXR* heterodimer that regulates genes expression [46]. Hence, we speculated that the methylation differences between these two genes are involved in their functions.

Lactation causes a massive metabolic demand in mammals due to which various homeostatic mechanisms are initiated, including a large increase in food intake. *NPY* expression may be involved in the physiological circumstances established during lactation that cause hyperphagia; the mRNA in the arcuate nucleus being significantly elevated during lactation [47]. On the other hand, suckling, which induces hyperprolactinemia, can stimulate the activation of hypothalamic *NPY* neurons, suggesting that *NPY* is modulated by prolactin (PRL) during lactation [48]. Increased *NPY* expression during lactation drives the chronic hyperphagia, and transmits the information to gonadotropin-releasing hormone (GnRH) neurons in order to suppress the GnRH neuronal activity, and thus control the luteinizing hormone levels (HL) [49].

The MCF-7 breast cancer cell line is a classic cell line of breast cancer, and is used in many breast cancer studies. The cells have some characteristics similar to those of differentiated mammary epithelial cells. Recently, studies have shown that epigenetic changes play an important role in the development of breast cancer, especially in DNAm abnormalities. Both human *PPARα* and *NPY* genes had a higher methylation level in the MCF-7 cells than that in the MCF-10A cells, which may be a concern with the cell type. This result combined with the lower methylation level of the goat *PPARα* gene in the lactation period than that in the dry period led us to speculate that the *PPARα* gene had a relatively low functional methylation level. The *NPY* gene methylation pattern provides a new insight to further investigate its function and mechanism.

In summary, this study revealed an integrated genome-wide DNAm difference and the distribution and proportion of four kinds of methylcytosines in the lactation and dry periods of goat mammary glands. A number of genes associated with mammary gland development and lactation process were found in DMRs. These findings provide essential information for the epigenetic regulation of the mammary gland in dairy goats.

## MATERIALS AND METHODS

### Ethics statement

This study was carried out in accordance with the recommendations of the Institutional Animal Care and Use Committee (IACUC) of Northwest A&F University (NWAFU-314020038), China. The protocol was reviewed and approved by the Institutional Animal Care and Use Committee (IACUC) of Northwest A&F University, China.

### Sample collection

The Xinong Saanen dairy goat is an excellent breed in China, and is mainly reared at the Xinong Saanen dairy goat breeding farm located at the Northwest A&F University, Yangling, Shaanxi, China. In total, four healthy female Xinong Saanen dairy goats were selected from the Yangling High-Tech Agriculture Demonstration Zone, Shaanxi Province, China. Mammary gland tissue samples were collected for RRBS sequencing. We cut a piece of mammary gland tissues at random from mammary gland. Two of the Xinong Saanen dairy goats were in the lactation period, which was classified as 15 days to six months postpartum (M1 and M2). The other two Xinong Saanen dairy goats were in the dry period, which was classified as 15 days after mammary involution (D1 and D2) [50, 51]. All samples were frozen in liquid nitrogen and stored at −80°C until use.

In order to verify the RRBS sequencinig results, we not only used the above-mentioned samples (M1, M2 and D1, D2) but also utilized the other samples. So, we collected four other Xinong Saanen dairy goat mammary gland tissue samples from a Xinong Sanen Dairy Goat Farm, Fuping, Shaanxi Province, China. Two mammary gland tissue samples from goats in the lactation period were named as M-I and M-II; two tissue samples from goats in the dry period were named as D-I and D-II, with each period having duplicate tissue samples. All samples were frozen in liquid nitrogen and stored at −80°C.

The MCF-7 cell line, a classic human breast cancer cell line, was cultured in our laboratory. The MCF-10A cells, human mammary epithelial cells, were kindly purchased by Stem Cell Bank, Chinese Academy of Science, Shanghai, China. Duplicate cells of both types of human cell lines (MCF-7-1, MCF-7-2, and MCF-10A-1, MCF-10A-2) were cultured for further study.

### RRBS library construction and sequencing

Methylation profiles of the mammary gland tissues were studied using the RRBS method by Guangzhou Gene Denovo Biotechnology Co. Ltd. (Guangzhou, China). Genomic DNA of mammary gland tissues (M1, M2, D1, D2) were isolated using the TIANamp Genomic DNA Kit. The qualified DNA was then digested by restriction endonucleases (*Msp*I) and repaired by 3′-end addition and adaptor ligation. The 250–500-bp fragments were then selected and treated with ZYMO EZ DNA Methylation-Gold kit for bisulfite conversion. The converted DNA was used for PCR amplification, and then the concentration was measured using an Agilent 2100 bioanalyzer instrument to construct the RRBS library, which was used for sequencing. The constructed library was subjected to high-throughput sequencing using the Illumina Hiseq 2500 platform with paired-end 125 bp sequencing (PE125).

### RRBS data analysis

Raw sequencing data were filtered and assembled. The low-quality reads (Q < 20 and N > 10%) were filtered out from the raw reads. High quality and clean reads were mapped to goat reference genomes using BSMAP (version 2.90) software by Guangzhou Gene Denovo Biotechnology Co. Ltd. (Guangzhou, China) [52]. In order to detect the level of DNAm at each base, a methylation site with a coverage greater than 10 and an average mass number greater than 20 were extracted for subsequent analysis. Only the unique mapped reads that had enzyme cutting sites could be used for standard analysis and personalized bioinformatic analysis. The unique mapped reads were analyzed to obtain methylation information of cytosine, including coverage analysis, methylation analysis, and DMRs analysis.

The methylation level was determined by dividing the number of reads covering each mC by the total reads covering that particular cytosine [53, 54], which was also equal to the mC/C ratio at each reference cytosine [30]. Only methylated cytosines with sequence depth coverage of at least 10, that were also were covered by at least four reads, were used.

### Identification of the important genes based on DMRs

A sliding-window approach was used to identify DMRs. All the DMR-related genes were clustered based on the Gene Ontology (GO) annotation database [53]. After multiple testing corrections, we choose to deem pathways with a Q value ≤ 0.05 as significantly enriched with DMR-related genes. Considering the significant enrichment to pathways, we found the main biochemical pathways and signal transduction pathways in which the DMR-related genes were located.

### Cell cultures

MCF-7 cells were grown in RPMI 1640 supplemented with 10.0% fetal bovine serum (FBS), 0.3 g/L glutamine, and 1.0% penicillin/streptomycin solution. Cells were maintained at 37°C, in a humidified atmosphere containing 5.0% CO_2_.

According to the culutre direction of Stem Cell Bank, Chinese Academy of Science, Shanghai, China, MCF-10A cells were cultured in MEGM kit Growth Media (Lonza/Clonetics, CC-3150) supplemented with 0.1% insulin, 0.4% FBS, 0.1% epidermal growth factor (EGF), 0.1% glucocorticoids, 0.1% cholera toxin and 1% penicillin/streptomycin solution. The cells were maintained at 37°C in a humidified atmosphere containing 5.0% CO_2_.

### DNA extraction and bisulfite conversion

Eight goat mammary gland tissue samples, as well as MCF-7 and MCF-10-A cells, were used for the bisulfite sequencing validation. The genomic DNA of MCF-7 and MCF-10A was isolated according to Takara MiniBEST Universal Genomic DNA Extraction Kit Ver. 5.0 (Takara Company, Dalian, China). The genomic DNA of the mammary gland tissue samples was extracted using phenol: chloroform, and treated with the EZ DNA Methylation-Gold kit (Zymo Research, Orange, CA) for bisulfite conversion. The genomic DNA was treated with bisulfite, which resulted in all of the non-methylated cytosine being converted to uracil, and the methylated cytosine remaining unchanged.

### Bisulfite sequencing validation of CpG islands for DMR-related genes

The CpG islands of goat *PPARα* and *RXRα* genes and human *PPARα* and *NPY* genes were predicted, and methylation primers were designed using the MethPrimer online software (http://www.urogene.org/methprimer/). The PCR products were purified and linked to the PGEM-T Easy Vector system (Promega Corp. WI, USA), then transformed to a *DH5α* competent cell in order to screen positive clones for sequencing.

In total, 15-20 positive clones were sequenced for each sample to identify the methylated CG sites. The methylation status of each CG locus was obtained by alignment using BioXM (version 2.6.0, developed by College of Agriculture, Nanjing Agricultural University, China). The methylated CG or unmethylated CG of the CpG islands was coded as 1/0. White-Black Rectangle of CG loci were delineated by MSR calculate (http://www.msrcall.com/) developed by our team (Lan’s and Liu’s group). Fisher’s exact test (χ^2^-test) was used to test for significance between the CpG islands, CG loci for both thelactation and dry period of dairy goats, and the MCF-7 and MCF-10-A cells of humans.

## SUPPLEMENTARY FIGURES AND TABLES











## Abbreviations

RRBS: reduced representation bisulfite sequencing
DNAm: DNA methylation
CDS: coding sequence
DMRs: differentially methylated regions
DNMT3: DNA (cytosine-5)-methyltransferase 3
PPARα: peroxisome proliferator-activated receptor alpha
NPY: neuropeptide Y
RXRα: retinoid X receptor alpha
GO: GeneOntology
