# Supplementary material for: Reduced representation bisulfite sequencing (RRBS) of dairy goat mammary glands reveals DNA methylation profiles of integrated genome-wide and critical milk-related genes
Source: Oncotarget. 2017 Dec 15;8(70):115326–44. doi: 10.18632/oncotarget.23260 (PMC5777775; doi:10.18632/oncotarget.23260)
Supplement: Supplementary file 1 [file oncotarget-08-115326-s001.pdf]

Reduced representation bisulfite sequencing (RRBS) of dairy goat mammary glands reveals DNA methylation profiles of integrated genome-wide and critical milk-related genes

SUPPLEMENTARY MATERIALS

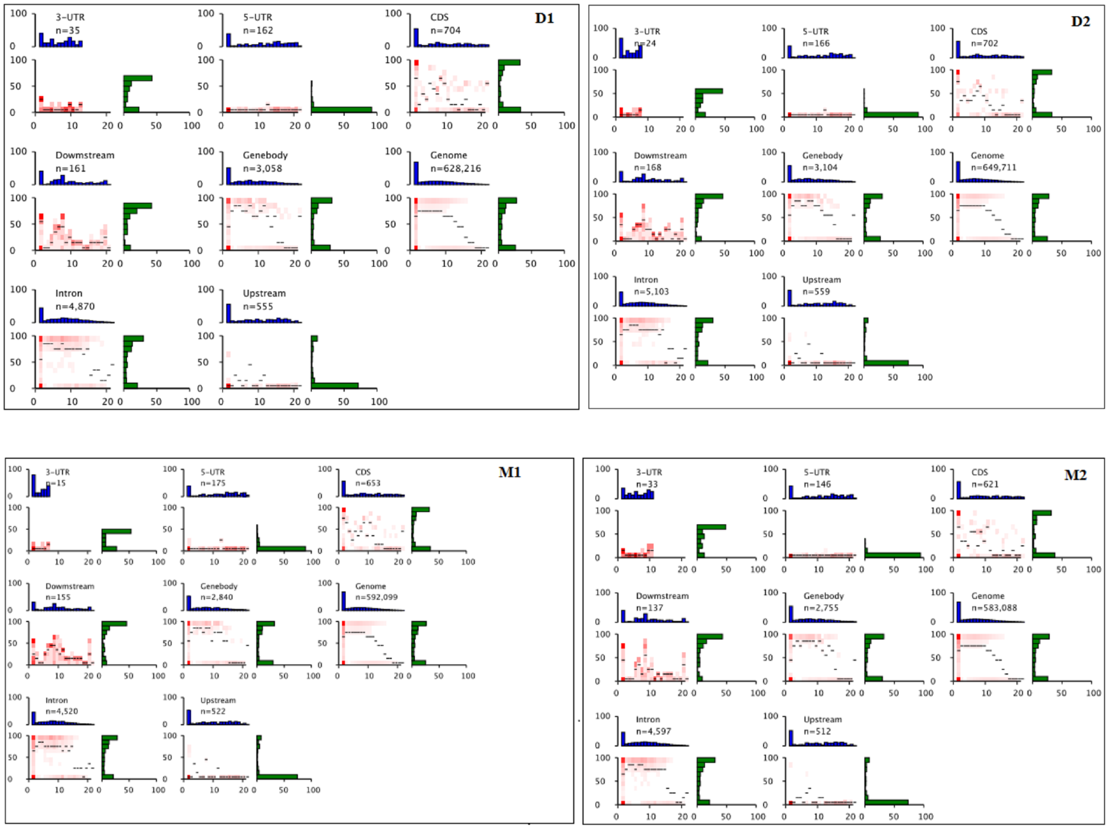

Supplementary Figure 1: Heatmaps show distinct methylation and CpG density patterns.
Note: Each panel represents a separate feature, and "n" refers to the numbers of analyzed CpGs (per-strand depth>=4) within that feature. CpG density (x-axis) is defined as numbers of CpG dinucleotides in 200 bp windows. Methylation level (y-axis) is defined as average methylation level of cytosines in CpGs. The thin black lines within each heat map denote the median methylation level of CpGs at the given local density. The red color gradient indicates abundance of CpGs that fall into bins of given methylation levels and CpG densities. The blue bar charts above each heat map show the distribution of CpG densities, projected onto the x-axis of the heat maps. The green bar charts to the right of the heat maps show the distribution of methylation levels, projected onto the y-axis of the heat maps.

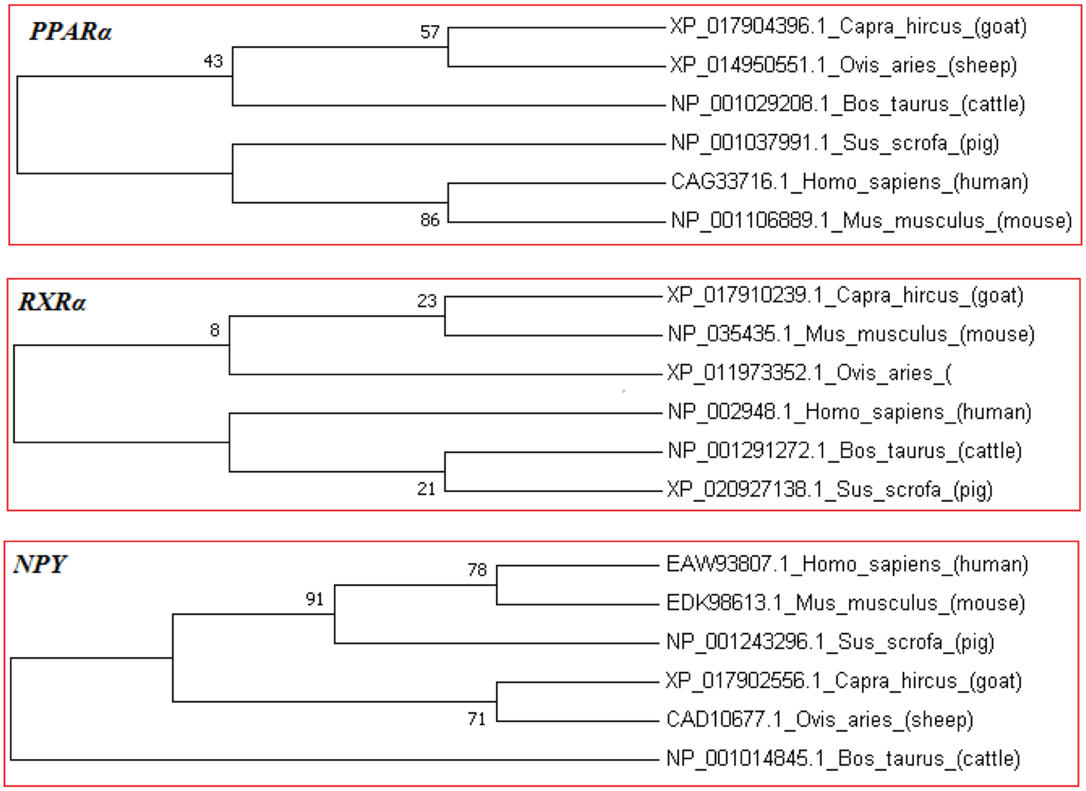

Supplementary Figure 2: Phylogenetic analysis of *PPARα*, *RXRα*and *NPY* genes.
Note: The bootstrap consensus evolutionary trees were constructed based on the amino acid sequences using MEGA7 soft Maximum Likelihood method. The numbers on the joints were bootstrap values of bootstrap tests.

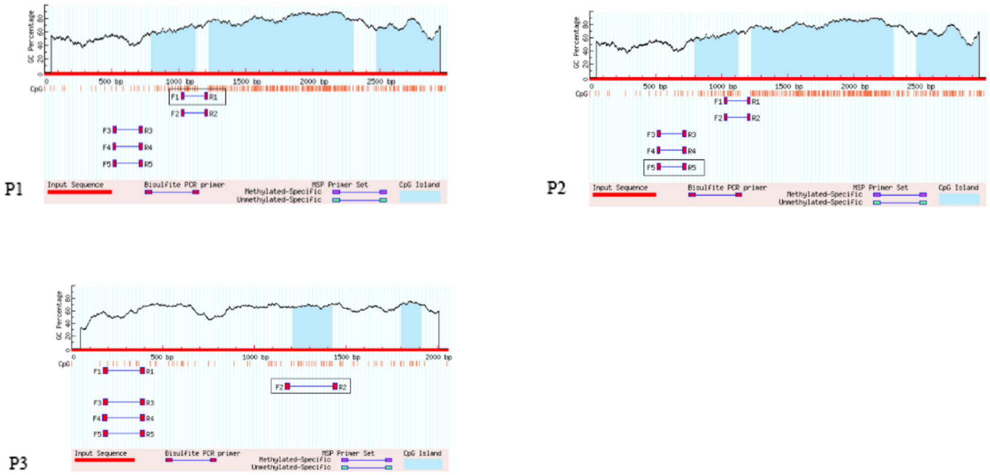

Supplementary Figure 3: CpG islands prediction and methylation primer design of goat *PPARα* gene.

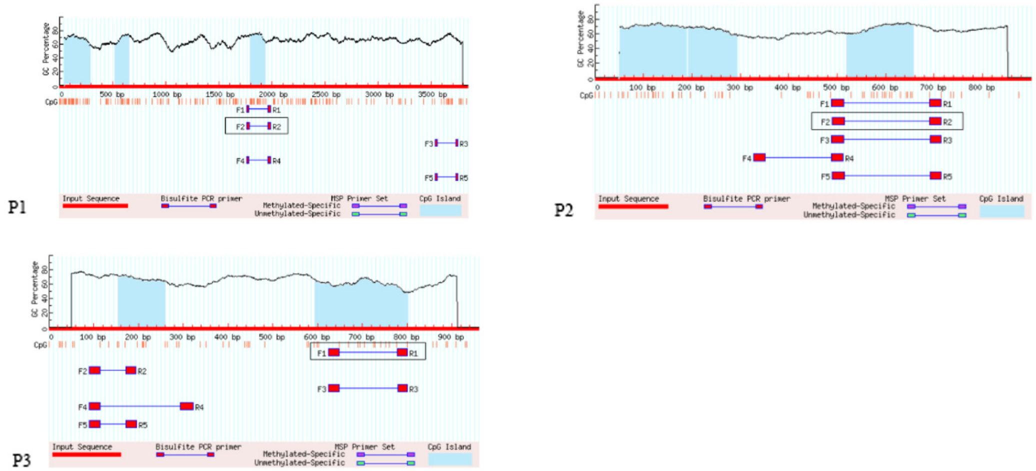

Supplementary Figure 4: CpG islands prediction and methylation primer design of goat *RXRα* gene.

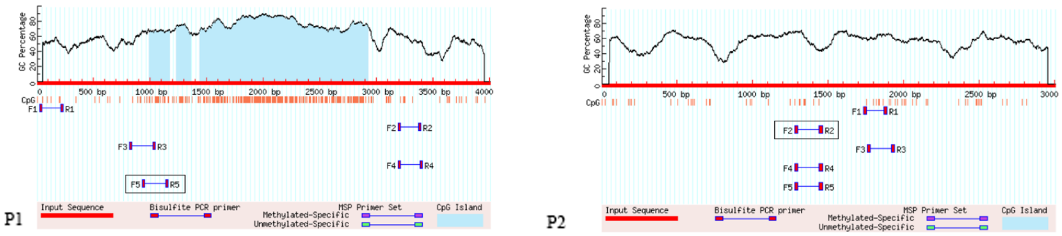

Supplementary Figure 5: CpG islands prediction and methylation primer design of human *PPARα* gene.

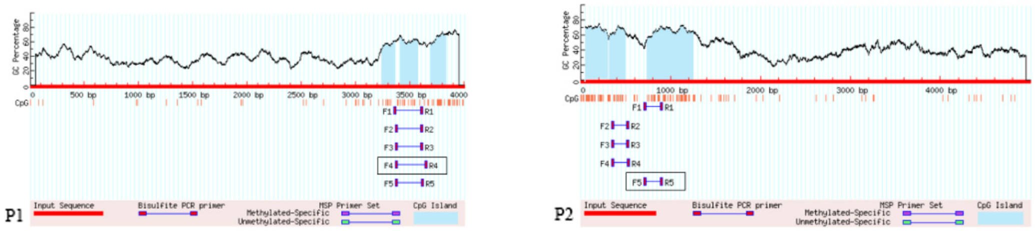

Supplementary Figure 6: CpG islands prediction and methylation primer design of human *NPY* gene.

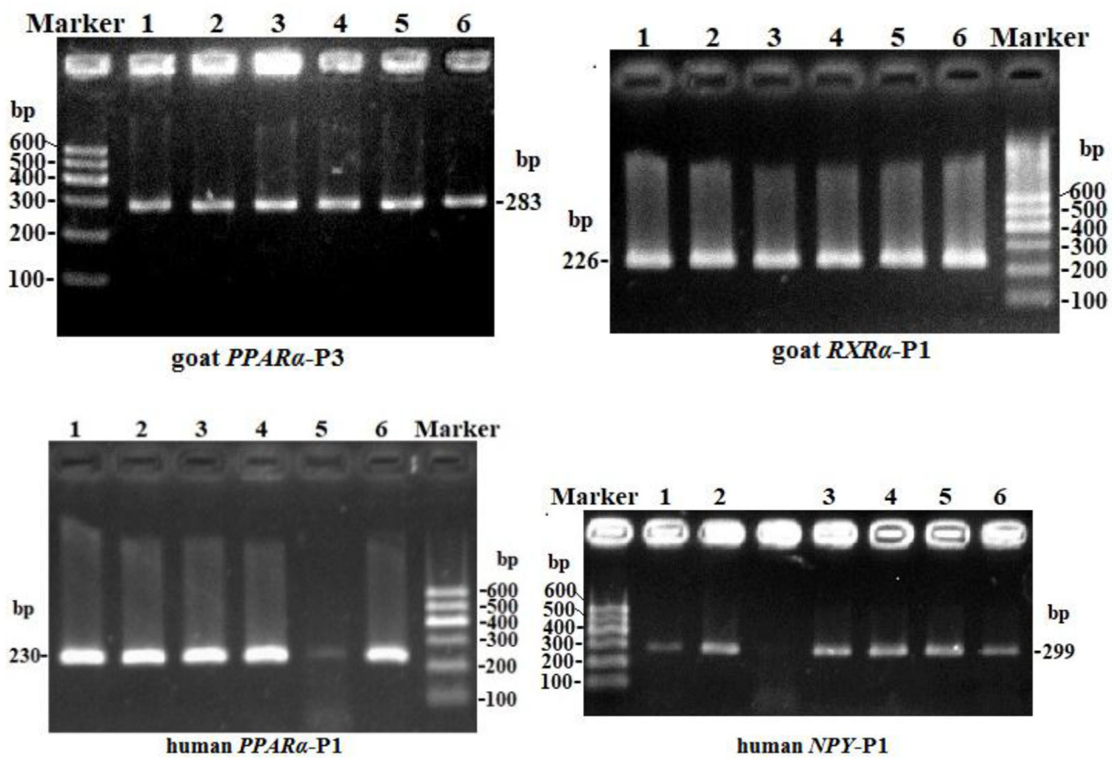

Supplementary Figure 7: Bacteria PCR electrophoresis diagram.

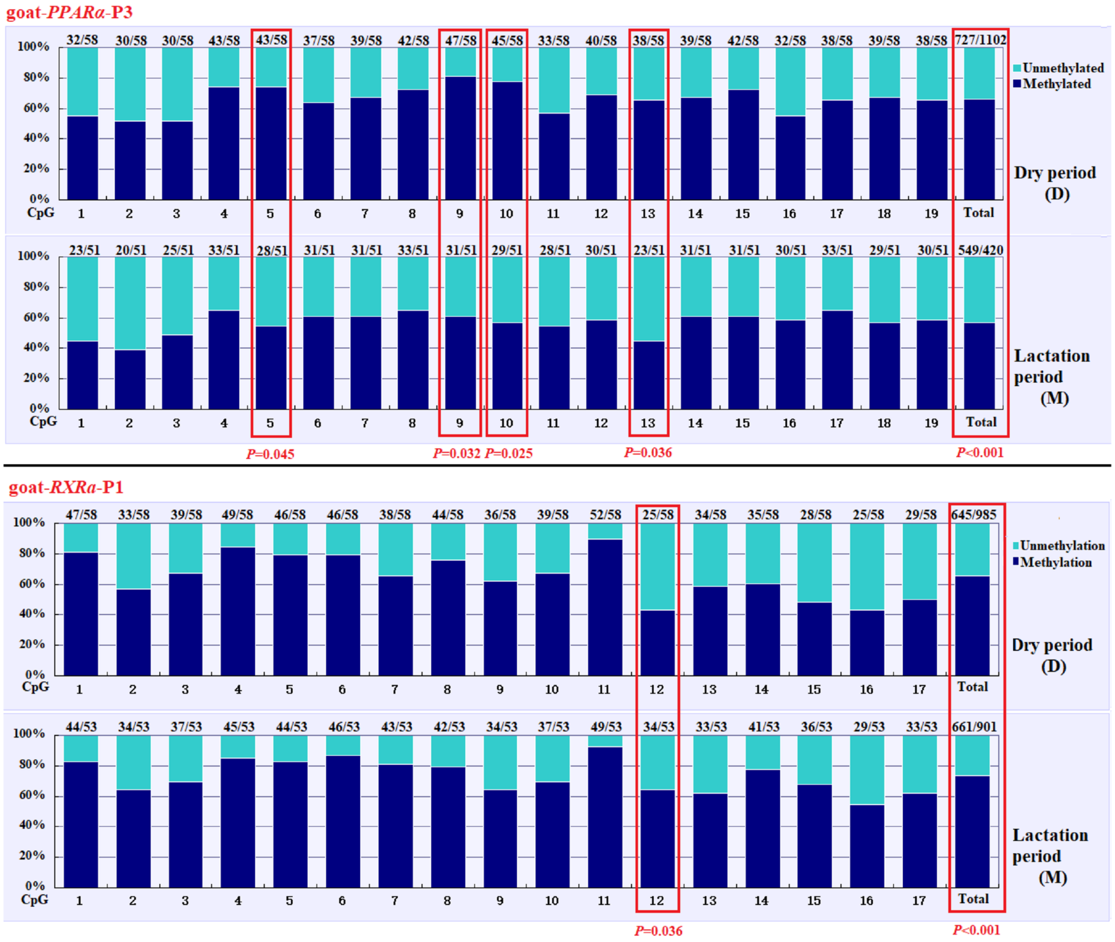

Supplementary Figure 8: Comparisons of DNA methylation differences for each CpG locus and total colonies of DMR within goat *PPARα* and *RXRα*genes between dry period and lactation period mammary glands.
Note: The **light blue bar** meant the number of the unmethylated cytosine, while the **dark blue bar** meant the number of the methylated cytosine; **X/Y** upon each bar meant the ratio of methylated cytosine versus total colonies; **Red frame** represented the significant difference between the two groups (P<0.05 or P<0.01).

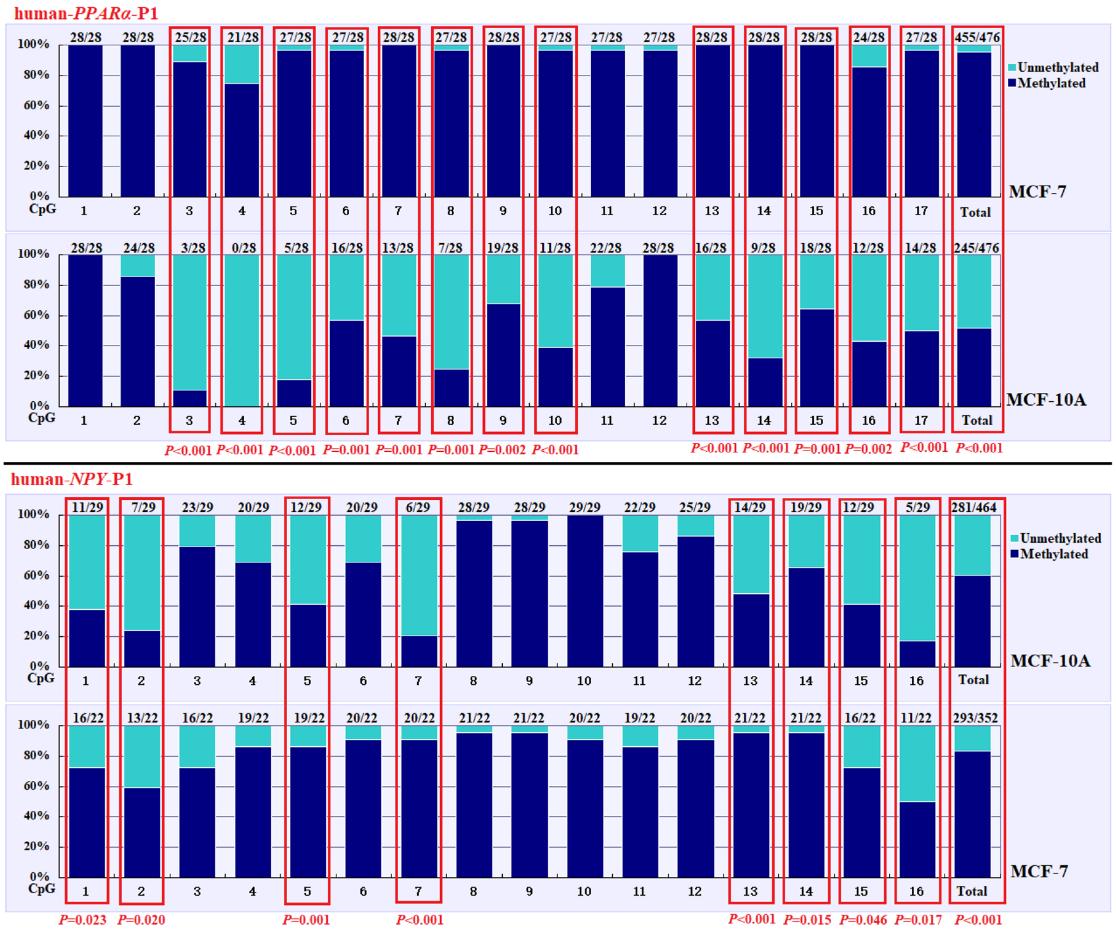

Supplementary Figure 9: Comparisons of DNA methylation differences for each CpG locus and total colonies of DMR within human *PPARα* and *NPY* genes between MCF-7 and MCF-10A cells.
Note: The **light blue bar** meant the number of the unmethylated cytosine, while the **dark blue bar** meant the number of the methylated cytosine; **X/Y** upon each bar meant the ratio of methylated cytosine versus total colonies; **Red frame** represented the significant difference between the two groups (P<0.05 or P<0.01).

Supplementary Table 1: Coverage percentage of CG, CHG and CHH.

See Supplementary file 1

Supplementary Table 2: Sequencing data information.

See Supplementary file 2

Supplementary Table 3: Methylation information of PPARα-P3, RXRα-P1 and NPY-P1 genes.

See Supplementary file 3

Supplementary Table 4: Methylation percentage of PPARα-P3, RXRα-P1 and NPY-P1 genes.

See Supplementary file 4
