# Supplementary material for: Reduced representation bisulfite sequencing (RRBS) of dairy goat mammary glands reveals DNA methylation profiles of integrated genome-wide and critical milk-related genes
Source: Oncotarget. 2017 Dec 15;8(70):115326–44. doi: 10.18632/oncotarget.23260 (PMC5777775; doi:10.18632/oncotarget.23260)
Supplement: Supplementary file 2 [file oncotarget-08-115326-s002.docx]

**Table 1: Coverage percentag**e **of CG, CHG and CHH**

|  | | CG | | | CHG | | | CHH | | |
| --- | --- | --- | --- | --- | --- | --- | --- | --- | --- | --- |
|  | | Total  Sites | Coverage Sites | Coverage Rate (%) | Total  Sites | Coverage  Sites | Coverage rate (%) | Total  sites | Coverage sites | Coverage  rate (%) |
| D1 | 3’-UTR | 43471 | 22560 | 51. 9 | 86041 | 47058 | 54.69 | 194672 | 98105 | 50.4 |
|  | 5’-UTR | 85354 | 46450 | 54. 42 | 94162 | 52068 | 55.3 | 187856 | 98687 | 52.53 |
|  | CDS | 465328 | 258350 | 55. 52 | 646210 | 374223 | 57.91 | 1291879 | 714081 | 55.27 |
|  | Intron | 1448519 | 711081 | 49. 09 | 2912399 | 1536503 | 52.76 | 6801989 | 3279667 | 48.22 |
|  | downstream2k | 163512 | 82701 | 50. 58 | 289251 | 153630 | 53.11 | 647144 | 318087 | 49.15 |
|  | upstream2k | 309839 | 158081 | 51.02 | 406162 | 214756 | 52.87 | 930911 | 453872 | 48.76 |
| D2 | 3’-UTR | 43471 | 23876 | 54.92 | 86041 | 50902 | 59.16 | 194672 | 110313 | 56.67 |
|  | 5’-UTR | 85354 | 47789 | 55.99 | 94162 | 54456 | 57.83 | 187856 | 105592 | 56.21 |
|  | CDS | 465328 | 266432 | 57.26 | 646210 | 390389 | 60.41 | 1291879 | 761404 | 58.94 |
|  | Intron | 1448519 | 753736 | 52.03 | 2912 399 | 1662064 | 57.07 | 6801989 | 3663080 | 53.85 |
|  | downstream2k | 163512 | 85889 | 52.53 | 289251 | 162971 | 56.34 | 647144 | 347261 | 53.66 |
|  | upstream2k | 309839 | 164901 | 53.22 | 406162 | 228775 | 56.33 | 930911 | 500295 | 53.74 |
| M1 | 3’-UTR | 43471 | 21681 | 49.87 | 86041 | 44483 | 51.7 | 194672 | 92812 | 47.68 |
|  | 5’-UTR | 85354 | 44003 | 51.55 | 94162 | 49243 | 52.3 | 187856 | 92398 | 49.19 |
|  | CDS | 465328 | 245650 | 52.79 | 646 210 | 354464 | 54.85 | 1291879 | 675566 | 52.29 |
|  | Intron | 1448519 | 675636 | 46.64 | 2912399 | 1455432 | 49.97 | 6801989 | 112134 | 45.75 |
|  | downstream2k | 163512 | 78309 | 47.89 | 289251 | 145142 | 50.18 | 647144 | 299323 | 46.25 |
|  | upstream2k | 309839 | 150650 | 48.62 | 406162 | 204019 | 50.23 | 930911 | 430247 | 46.22 |
| M2 | 3’-UTR | 43471 | 21681 | 49.87 | 86041 | 45868 | 53.3 | 194672 | 97262 | 49.96 |
|  | 5’-UTR | 85354 | 44510 | 52.15 | 94162 | 50140 | 53.25 | 187856 | 94703 | 50.41 |
|  | CDS | 465328 | 244030 | 52.44 | 646210 | 356534 | 55.17 | 1291879 | 685738 | 53.08 |
|  | Intron | 1448519 | 682084 | 47.09 | 2912399 | 1500683 | 51.53 | 6801989 | 3237780 | 47.6 |
|  | downstream2k | 163512 | 78679 | 48.12 | 289251 | 148945 | 51.49 | 647144 | 310272 | 47.94 |
|  | upstream2k | 309839 | 151511 | 48. 9 | 406162 | 208249 | 51.27 | 930911 | 442461 | 47.53 |

**Note:** **Note: D1** and **D2** are the samples of goat dry period mammary gland; **M1** and **M2** are the samples of goat lactation period mammary gland; H represents non-C base.
